# Intravitreal panitumumab and retinal pigment epithelium proliferation in laser-induced retinal degeneration in rabbits

**DOI:** 10.3389/fopht.2025.1641194

**Published:** 2025-08-07

**Authors:** Mukharram M. Bikbov, Gyulli M. Kazakbaeva, Songhomitra Panda-Jonas, Iskander D. Valishin, Aigul M. Nizamutdinova, Jost B. Jonas

**Affiliations:** ^1^ Ufa Eye Research Institute, Bashkir State Medical University, Ufa, Russia; ^2^ Department of Ophthalmology, Medical Faculty Heidelberg, Heidelberg University, Heidelberg, Germany; ^3^ L V Prasad Eye Institute, Hyderabad, Telangana, India; ^4^ Privatpraxis Prof Jonas und Dr Panda-Jonas, Heidelberg, Germany; ^5^ Rothschild Foundation Hospital, Institut Français de Myopie, Paris, France; ^6^ Singapore Eye Research Institute, Singapore National Eye Center, Singapore, Singapore; ^7^ Beijing Visual Science and Translational Eye Research Institute (BERI), Beijing Tsinghua Changgung Hospital, Tsinghua Medicine, Tsinghua University, Beijing, China; ^8^ Department of Ophthalmology and Visual Sciences, The Chinese University of Hong Kong, Hong Kong, Hong Kong SAR, China; ^9^ New York Eye and Ear Infirmary of Mount Sinai, Icahn School of Medicine at Mount Sinai, New York, NY, United States

**Keywords:** panitumumab, epidermal growth factor receptor inhibitor, macular degeneration, retinal pigment epithelium, macular scar, intravitreal medication

## Abstract

**Purpose:**

This study aims to examine the effect of intravitreally applied epidermal growth factor (EGF) receptor blocker panitumumab on the proliferation of retinal pigment epithelium cells (RPE) in an experimental model of localized retinal degeneration.

**Methods:**

The experimental study included rabbits with age of 2 to 3 months and body weight of 2.5–3 kg and which were randomly distributed into a study group and control group. The right eyes received two retinal argon laser coagulation spots (500 mW; diameter, 100 μm; duration, 0.5 s), applied with an interval of 2 min at the same location close to the vascular streak in the posterior fundus region. For five times at 2-day intervals, the rabbits of the study group received intravitreal injections of 1 mg panitumumab (0.10 mL), and the rabbits of the control group had intravitreal injections of 0.10 mL Ringer’s solution. At baseline, at each time point of re-examination, and at study end, the animals were examined by fundus photography and optical coherence tomography of the laser spot.

**Results:**

The study included 19 rabbits (study group: 10 animals; control group: nine animals). After the third injection and at study end, the laser-induced area of depigmentation + hyperpigmentation combined did not vary significantly between the study group and the control group (1.43 ± 0.63 mm^2^ versus 1.63 ± 0.77 mm^2^; *P* = 0.56; and 1.37 ± 0.63 mm^2^ versus 1.61 ± 0.74 mm^2^; *P* = 0.46, respectively). At the same time points, the area with hyperpigmentation was significantly smaller in the study group than in the control group (0.16 ± 0.15 mm^2^ versus 0.80 ± 0.59 mm^2^; *P* = 0.01; and 0.14 ± 0.14 mm^2^ versus 0.70 ± 0.56 mm^2^; *P* = 0.02, respectively). At the same time points, the ratio of the hyperpigmented area to the combined depigmented + hyperpigmented area was significantly smaller in the study group than in the control group (0.11 ± 0.09 versus 0.43 ± 0.19 mm^2^; *P* < 0.001; and 0.10 ± 0.08 versus 0.35 ± 0.23mm^2^; *P* = 0.006, respectively)

**Conclusions:**

These findings indicate that intravitreally administered panitumumab was associated with reduced subretinal hyperpigmentation in a laser-induced model of retinal injury. While this may reflect a modulation of the RPE response, including the potential suppression of RPE proliferation, further studies incorporating histological and molecular analyses are warranted to confirm its effect on subretinal fibrosis.

## Introduction

Exudative age-related macular degeneration (AMD) and exudative myopic macular degeneration belong to the most common causes of irreversible vision impairment and blindness worldwide (1, 2). At the end stages of AMD and myopic macular degeneration, they are characterized by neovascularization extending from the choroid into the space beneath the retinal pigment epithelium (RPE) and beneath the retina and by subretinal proliferation of RPE cells (3–5). Since the clinical introduction of intravitreally injected vascular endothelial growth factor (VEGF) antibodies, such as bevacizumab, ranibizumab, aflibercept, and faricimab, the neovascular part of these maculopathies has been addressed. It results in a temporary increase in visual acuity and an overall slowing of the process of vision deterioration (6–8). The RPE-dependent proliferative part of exudative AMD and myopic macular degeneration has not markedly been addressed so far. The subfoveal RPE proliferation is characterized by a fibrous pseudo-metaplasia of the macular RPE cells, which eventually form a dense subretinal scar (9, 10). This scar separates the foveal photoreceptors from the nourishing foveal choriocapillaris and is a major cause for the irreversible loss of central vision in exudative macular degenerations (5, 9, 10). Experimental studies have revealed that the RPE cells have receptors for epidermal growth factor (EGF) and that EGF is a strong promoter of RPE cell proliferation and migration, while, vice versa, EGF receptor blockers inhibit the proliferation and migration of RPE cells (11–19). Correspondingly, a clinical study suggested that eyes with exudative/neovascular AMD or exudative myopic maculopathy have an increased intraocular concentration of EGF (20, 21). We therefore examined whether an EGF receptor antibody, here panitumumab, reduces the RPE proliferation in an experimental model of subretinal RPE proliferation.

## Methods

The experimental study included rabbits (gray Soviet chinchilla; all male; age, 2 to 3 months; weight, 2.5–3 kg). All animals were treated in accordance with the ARVO (Association for Research in Vision and Ophthalmology) Statement for the Use of Animals in Ophthalmic and Vision Research. The Ufa Eye Research Institute Biomedical Ethics Committee approved the investigation which is reported in accordance with the ARRIVE (Animal Research: Reporting of *In Vivo* Experiments) guidelines. All methods were performed in accordance with the relevant guidelines and regulations. The animals were purchased from a commercial vendor (Federal State Unitary Enterprise ‘‘Scientific and Production Association for Immunological Preparations ‘‘Microgen’’ of the Ministry of Health of the Russian Federation, Ufa, Bashkortostan, Russia) and were kept at a constant temperature (22 ± 1°C) in a light-controlled environment (lights on from 7 a.m. to 7 p.m.) with *ad libitum* access to food and water.

The right eyes of the rabbits from the study group underwent laser-induced retinal coagulation close to the vascular streak in the posterior fundus region by applying an argon laser coagulation (wavelength, 532 µm) spot at 500 mW, with a spot diameter of 100 μm and a duration of 0.5 s twice with an interval of 2 min at the same location. The laser beam was irradiated on the retina using indirect ophthalmoscopic lens (78 diopters) normally taken for ophthalmoscopy.

Immediately after the laser application, the rabbits of the study group received an intravitreal injection of 1 mg of panitumumab in 0.10 mL. The right eyes of the rabbits from the control group received an intravitreal injection of 0.10 mL of Ringer’s solution (Gematek OOO Company, Moscow, Russia). Directly after each injection, we measured the intraocular pressure (IOP) of the right eyes. The left eyes remained untouched. The injections and the retinal laser coagulation, carried out at the same setting, were performed in general anesthesia which was achieved by an intramuscular injection (biceps femoris) of Zoletil (15 mg/kg) (tiletamine mixed with zolazepam; Valdepharm Co., Val-de-Reuil, France) and xylazine (20 mg/kg) (Xyla; Interchemie Werken, De Adelaar B.V., A Waalre, The Netherlands). We additionally applied anesthetic eye drops (0.4% oxybuprocain, Inocain; Promed Exports, New Delhi, India) topically. The injections were carried out in the temporal upper quadrant of the globe at a distance of 3 to 4 mm from the limbus. The technique has been described recently in detail (22, 23).

**Figure 1:**
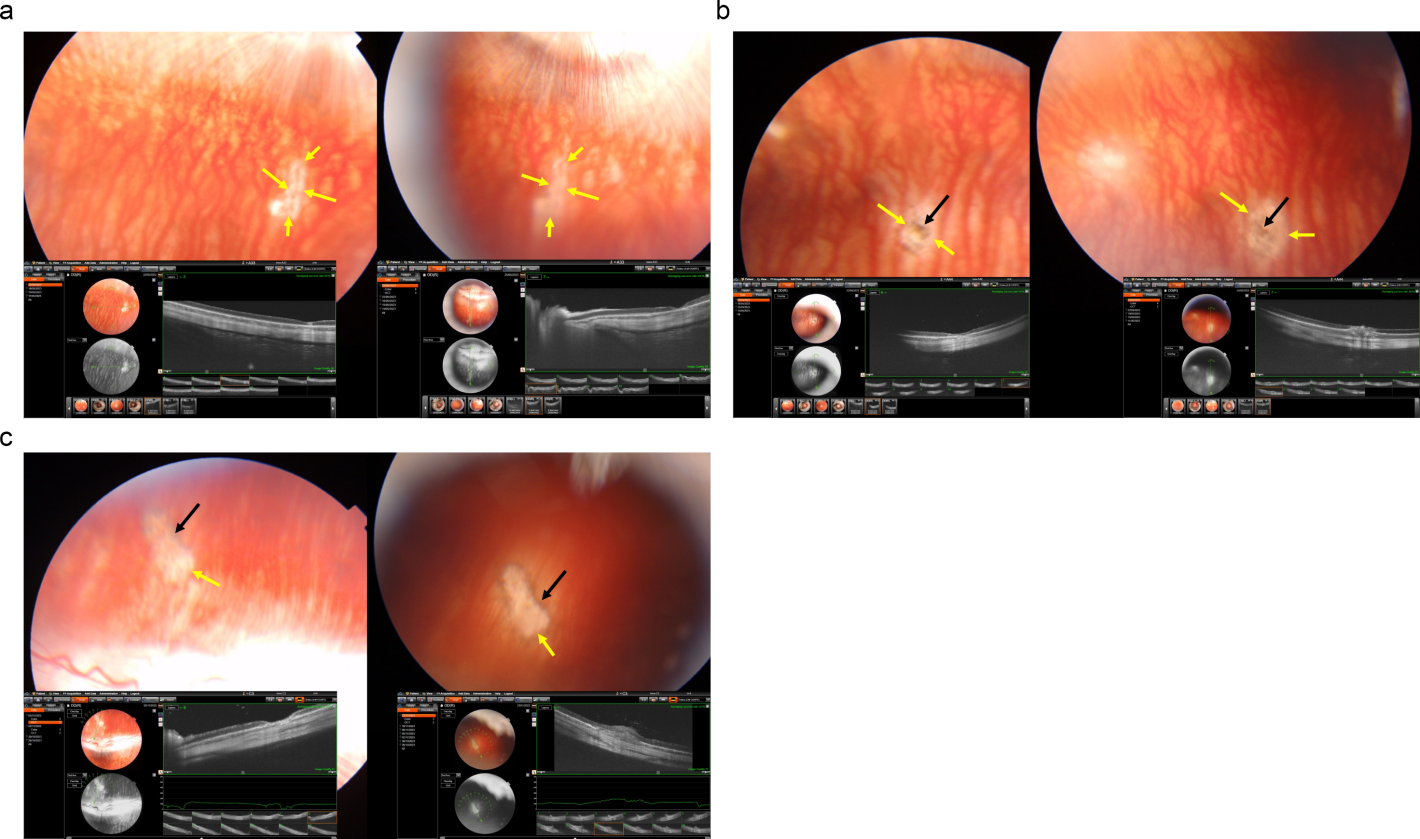
Fundus photographs and optical coherence tomographic images of three rabbits **(a-c)** with a laser-induced depigmentation and hyperpigmentation, and additionally receiving intravitreal injections of panitumumab; the images were taken after the third injection (left part of each **a-c**) or at study end (right part of the images); yellow arrows: depigmented area; black arrows: pigmentations.

**Figure 2:**
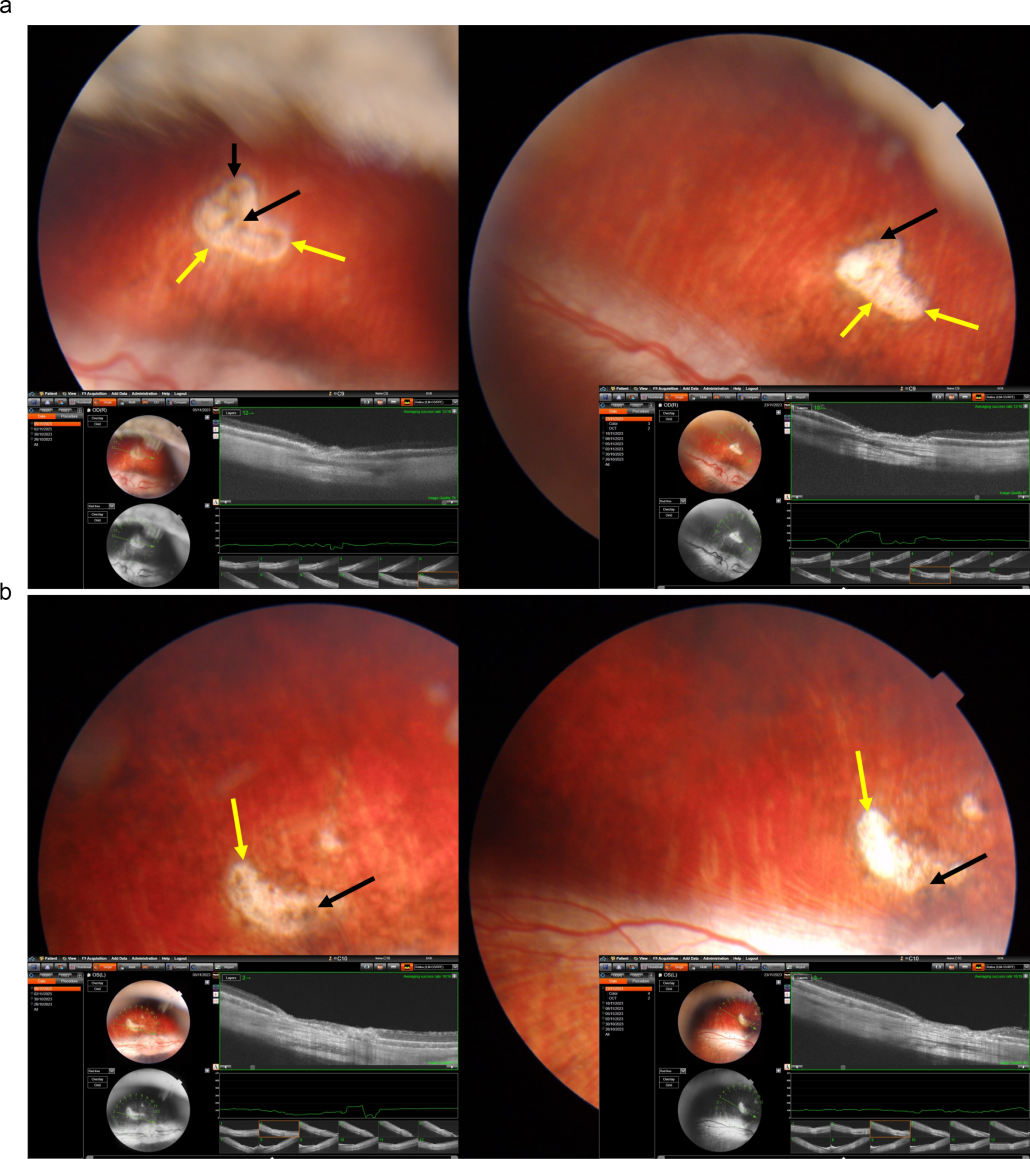
Fundus photographs and optical coherence tomographic images of two rabbits **(a, b)** with a laser-induced depigmentation and hyperpigmentation, and additionally receiving intravitreal injections of Ringer’s solution; the images were taken after the third injection (left part of each **a, b**) or at study end (right part of the images); yellow arrows: depigmented area; black arrows: pigmentations.

The injections of panitumumab and of Ringer´s solution into the right eyes were repeated four times in intervals of 2 days (i.e., in total, five injections per eye and animal). At baseline, at the time points of the re-injections, and at study end 3 days after the last (fifth) injection or 13 days after baseline, the animals were re-examined by inspection of the external eye, non-contact tonometry (PT 100 Portable Non-Contact Tonometer, Reichert Co, Depew/Buffalo, NY, USA), fundus photography, and optical coherence tomography (SS-OCT (DRI-OCT, Triton; Topcon Inc., Tokyo, Japan) of the laser spot (**Figures 1**, **2**). At 14 days after the last injection, the rabbits were sacrificed under deep general anesthesia (induced by an intramuscular injection of xylazine (3 mg/kg), ketamine (25 mg/kg), and zolazepamum (32 mg/kg) by injecting magnesium sulfate at 4 mL (1 g) and lidocaine at 5 mL (50 mg) into an ear vein.

Using morphometry of the fundus photographs and on the OCT images, we measured the area of the depigmented region and the area of the hyperpigmented region. The latest examination was performed at study end at 13 days after baseline, i.e., after the laser application and the concomitant first intravitreal injections.

Using a statistical software package (SPSS for Windows, version 27.0, IBM-SPSS, Chicago, IL, USA), we determined the mean values ± standard deviations of the main outcome parameters, i.e., the size of the depigmented region in the region of the laser spot application, and the size of the area with hyperpigmentation within the laser spot region. We applied Wilcoxon–Mann–Whitney test for unpaired samples to compare the parameters between the study and the control group. A two-sided *P*-value was considered statistically significant if it was smaller than 0.05.

## Results

**Figure 3:**
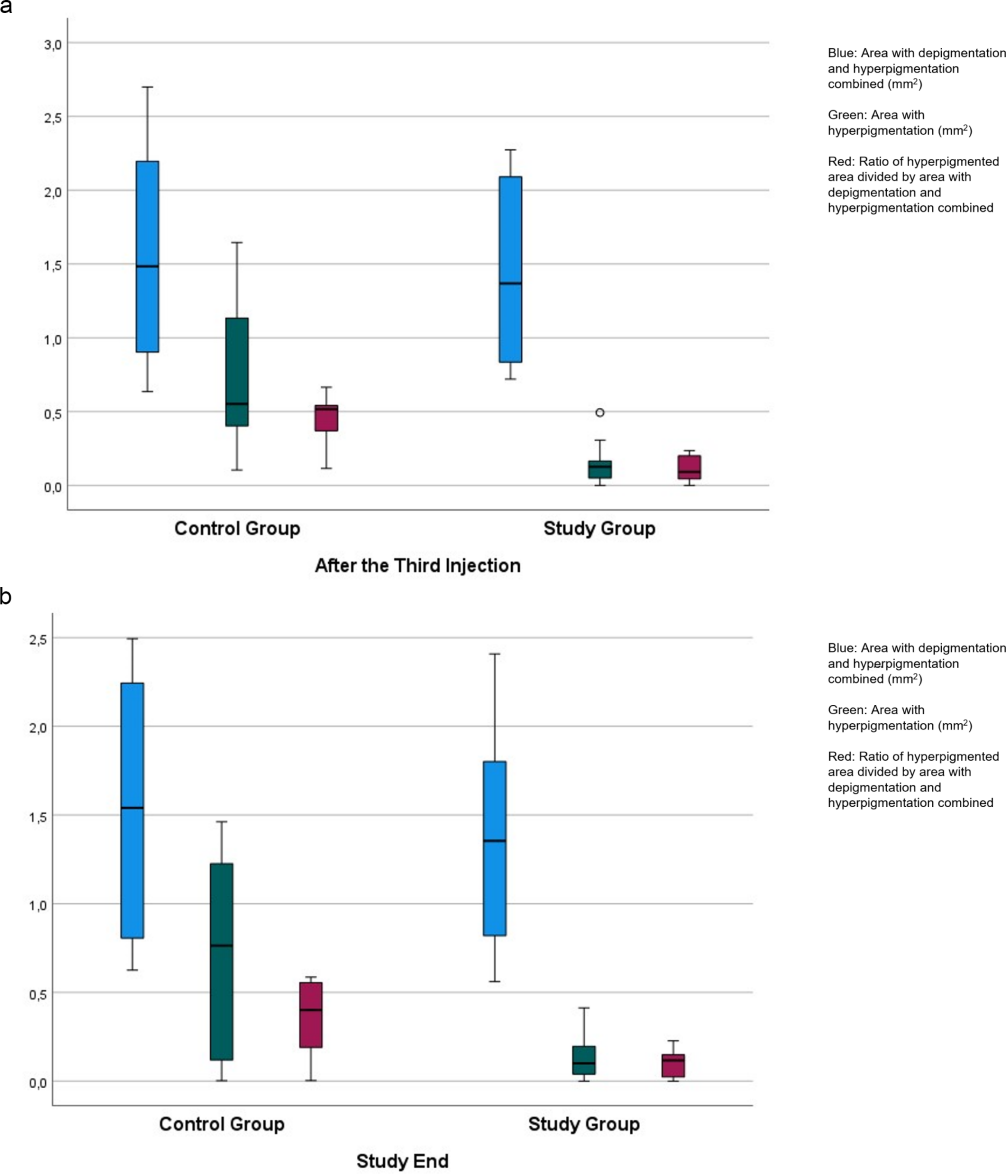
Boxplots showing the distribution of the area with hyperpigmentation combined with the area with depigmentation, the area with hyperpigmentation only, and of the ratio of the hyperpigmented area divided by the area with hyperpigmentation combined with the area with depigmentation in rabbits with laser-induced retinal degeneration, additionally receiving intravitreal injections of panitumumab (study group) or Ringer´s solution (control group), examined after the third injection **(a)**, and taken at study end **(b)**.

The study included 19 rabbits assigned to a study group of 10 animals and a control group of nine animals. After the third injection, the area of the laser-induced region of depigmentation and hyperpigmentation combined was 1.43 ± 0.63 mm^2^ in the study group and 1.63 ± 0.77 mm^2^ in the control group without a significant difference between both groups (*P* = 0.56). The area with hyperpigmentation was significantly smaller in the study group than in the control group (0.16 ± 0.15 mm^2^ versus 0.80 ± 0.59 mm^2^; *P* = 0.01) (**Figure 3**). The ratio of the hyperpigmented area to the combined depigmented and hyperpigmented area was significantly smaller in the study group than in the control group (0.11 ± 0.09 versus 0.43 ± 0.19 mm^2^; *P* < 0.001) (**Table 1**) (**Figure 3**).

**Table 1:** Measurements of the area of laser-induced depigmentation and hyperpigmentation in rabbits, additionally receiving an intravitreal injection of panitumumab (study group) or Ringer’s solution (control group).

Number	Study group/control group	Depigmented area at mid-study (mm^2^)	Hyperpigmented area at mid-study (mm^2^)	Ratio of depigmented area/hyperpigmented area at mid-study	Depigmented area at study end (mm^2^)	Hyperpigmented area at study end (mm^2^)	Ratio of depigmented area/hyperpigmented area at study end
А1	Study group	1.21	0.13	0.11	1.21	0.16	0.13
А2	Study group	0.73	0.17	0.23	0.86	0.09	0.10
А3	Study group	0.72	0.12	0.17	0.56	0.08	0.14
A33	Study group	0.91	0.00	0.00	0.78	0.00	0.00
A44	Study group	1.53	0.31	0.20	1.50	0.23	0.16
А5	Control group	0.90	0.10	0.12	0.79	0.00	0.00
А6	Control group	0.64	0.12	0.18	0.81	0.01	0.01
А7	Control group	0.74	0.40	0.54	0.63	0.12	0.19
А8	Control group	2.13	1.10	0.52	2.21	1.23	0.55
C1	Study group	2.20	0.10	0.04	Not measurable	Not measurable	Not measurable
C2	Study group	2.27	0.05	0.02	2.41	0.11	0.05
C3	Study group	2.09	0.49	0.24	1.82	0.41	0.23
C4	Study group	1.85	0.13	0.07	1.79	0.00	0.00
C5	Study group	0.84	0.05	0.06	Not measurable	Not measurable	Not measurable
C6	Control group	1.45	0.54	0.37	1.54	0.55	0.36
C7	Control group	2.70	1.65	0.61	2.24	1.26	0.56
C8	Control group	1.48	0.55	0.37	1.53	0.76	0.50
C9	Control group	2.20	1.13	0.52	2.30	0.92	0.40
C10	Control group	2.42	1.61	0.66	2.49	1.46	0.59

At study end, the area of depigmentation and hyperpigmentation combined was 1.37 ± 0.63 mm^2^ in the study group and 1.61 ± 0.74 mm^2^ in the control group without a significant difference between both groups (*P* = 0.46). The area with hyperpigmentation was significantly smaller in the study group than in the control group (0.14 ± 0.14 mm^2^ versus 0.70 ± 0.56 mm^2^; *P* = 0.02) (**Figure 3**). The ratio of the hyperpigmented area to the combined depigmented and hyperpigmented area was significantly smaller in the study group than in the control group (0.10 ± 0.08 versus 0.35 ± 0.23 mm^2^; *P* = 0.006) (**Figure 3**).

The IOP did not change significantly (all *P* < 0.05) during the study period.

## Discussion

In this experimental study on rabbits, eyes receiving intravitreal panitumumab injections as compared to eyes receiving intravitreal Ringer´s solution injections showed a significantly smaller hyperpigmented area in the laser-induced lesion, while the size of the laser-induced spot of depigmentation plus the area with hyperpigmentation combined did not vary significantly between both groups (**Figure 3**). The findings suggest that the laser application led to a comparable size of the depigmented combined hyperpigmented lesion, while hyperpigmentation alone, presumably corresponding to proliferated RPE cells, was less marked when panitumumab was intravitreally injected.

The results of our study cannot be directly compared with observations made in other studies since the influence of an intravitreally applied EGF receptor blocker such as panitumumab on RPE cell proliferation *in vivo* has not been examined yet.

The subretinal proliferation of RPE cells in neovascular maculopathies, namely, in exudative AMD, polypoidal vascular choroidopathy (PVC), and macular neovascularization as part of pathologic myopia, has only been rarely discussed and addressed in previous investigations. Besides the exudative part with a subretinal, intraretinal, or sub-RPE edema, scar formation by proliferating RPE cells in the foveal region is a major and irreversible cause for vision loss in these disorders. Histological studies have shown that the subretinal scars, also called subretinal membranes, are formed by RPE cells which, albeit irregularly arranged and irregularly pigmented, still have contact with a basal membrane. They thus fulfill the condition of a fibrous pseudo-metaplasia of RPE cells (10). One may discuss that the choroidal neovascularization breaking through the barrier of Bruch´s membrane into the sub-RPE space may be the primary event, which, by additional growth factors such as EGF, stimulates RPE cells to migrate and proliferate. Accordingly, clinical studies have shown an increased intraocular concentration of various growth factors, including EGF, in eyes with exudative/neovascular AMD and in eyes with pathologic myopia and macular neovascularization (20, 21). Based on these findings obtained in clinical and experimental studies, one may infer that the therapeutic inhibition of EGF in eyes with unwanted RPE proliferations may be an option to prevent or reduce the development of a subretinal scar formation.

The results of the present investigation support that notion since the animals with intravitreal panitumumab injections as compared to those with intraocular injections of Ringer´s solution showed a significantly smaller region with hyperpigmentation, while the size of the area with laser-induced RPE damage (i.e., the combined depigmented and hyperpigmented area) did not differ significantly between both animal groups. In view of the intraocular tolerability of intravitreally administered panitumumab, as suggested in previous investigations, one may consider to discuss the clinical application of a combined therapy of anti-VEGF drugs and EGF-receptor blockers in eyes with neovascular maculopathy (19, 22–24).

While the finding that the hyperpigmented areas were significantly reduced in panitumumab-treated eyes may be noteworthy and may perhaps suggest a possible inhibitory effect on RPE proliferation, one must clearly bear in mind that caution has to be applied against directly equating a reduction in pigmentation with suppression of fibrotic changes or RPE proliferation. The hyperpigmentation observed in fundus imaging may reflect the presence of melanin-laden RPE cells, but it does not necessarily indicate active cell proliferation, nor does it confirm the presence or absence of fibrous metaplasia. To substantiate a claim that panitumumab might reduce RPE proliferation and subretinal fibrosis, additional complementary examinations are necessary, including immunohistochemical assessment of fibrosis and epithelial mesenchymal transition, histological examinations for extracellular matrix deposition and fibroblastic morphology, and molecular analyses of an epithelial–mesenchymal transition. Without having conducted these additional investigations, the reduction in hyperpigmentation as observed in the present study may conservatively be interpreted as a surrogate for an altered RPE response to the lesion-induced injury rather than as an evidence of reduced fibrotic transformation.

Other limitations of the present investigation may also be discussed. First, as for any experimental animal study, species differences between humans and rabbits have to be taken into account. Panitumumab is a fully human monoclonal IgG2-antibody which is produced by recombinant DNA technology in a mammal cell line (25). Its activity to block the EGF-receptor in rabbits will be lower than in humans; thus, if applied in patients, the effect of panitumumab may be higher than observed in the present study. It may be considered that in various previous studies human EGF was cross-reactive in several other species, including rabbits (26–29). Second, species differences between rabbits and men in the anatomy and physiology of the macula prevent a direct transfer of the findings made in the rabbits of our study onto the clinical situation. Third, the study duration was relatively short, so observations on the long-term effect were not made. Fourth, the study did not assess the compatibility of a combined application of panitumumab and an VEGF-antibody such as ranibizumab, while in a clinical setting, both drugs are supposed to be given at the same setting. Fifth, we did not conduct dose–response studies. Sixth, we measured the IOP under general anesthesia, which may have influenced the IOP. Since the IOP did not change significantly during the study period, it is unlikely that this potential imitation of the study had an influence on the main findings of the study.

In conclusion, this experimental study on rabbits with a laser-induced damage of the RPE showed that eyes with a repeatedly intraocularly applied EGF-receptor blocker, i.e., panitumumab, showed a smaller hyperpigmented area, which, if taken as a surrogate for the proliferating RPE cells, suggests that the intraocularly applied panitumumab was helpful to reduce an unwanted subretinal proliferation of RPE cells in a model of laser-induced macular degeneration.

## Data Availability

The raw data supporting the conclusions of this article will be made available by the authors, without undue reservation.
